# Supplementary material for: Deformed wing virus type A, a major honey bee pathogen, is vectored by the mite Varroa destructor in a non-propagative manner
Source: Sci Rep. 2019 Aug 27;9:12445. doi: 10.1038/s41598-019-47447-3 (PMC6712216; doi:10.1038/s41598-019-47447-3)

Supplementary Information

Title:

Deformed wing virus type A, a major honey bee pathogen, is vectored by the mite Varroa destructor in a non-propagative manner.

Authors:

Francisco Posada-Florez*, Anna K. Childers, Matthew C. Heerman, Noble I. Egekwu, Steven C. Cook, Yanping Chen, Jay D. Evans, Eugene V. Ryabov*

Affiliation:

USDA, Agricultural Research Service, Bee Research Laboratory, Beltsville Agricultural Research Center, 10300 Baltimore Avenue, Bldg. 306, Beltsville MD 20705, USA

This PDF file includes:

Supplementary Table 1. Primers used in this study.

Supplementary Table 2. Detection of honey bee mRNA in *Varroa* mites by RT-qPCR.

Supplementary Table 3. Detection and quantification of honey bee mRNAs in RNAseq libraries from phoretic *Varroa* mites following starvation or feeding.

Supplementary Table 4. Detection of honey bee mRNAs in RNAseq libraries from different *Varroa* mite developmental stages.

Supplementary Figure S1. Alignments of consensus DWV RNA nucleotide sequences from honey bees and mites in Experiment 2: “*Varroa*-mediated DWV transmission experiment”.

Supplementary Figure S2. DWV polymorphisms in honey bees and mites in Experiment 2: “*Varroa*-mediated DWV transmission experiment”.

Supplementary Figure S3. Single stranded RNA degradation in disrupted honey bee hemolymph cells.

Supplementary Table 1. Primers used in this study.

Primer ID	Nucleotide sequence (5' to 3')	Target: GenBank accession number, (position in the nucleotide sequence), polarity	Applications
**DWV-qPCR-F**	GAGATTGAAGCGCATGAACA	AY292384, (6471- 6490), sense	DWV RT-qPCR
**DWV-qPCR-R**	tgaattcagtgtcgcccata	AY292384, (6600 - 6581), complement	DWV RT-qPCR
**Varr-qbACT-R**	TGAAGGTAGTCTCATGGATAC	XR_002672520 , (142 - 122) complement	Varroa actin mRNA RT-qPCR
**Varr-qbACT-F**	GTCTCTGTTCCAGCCCTCGTTC	XR_002672520, (81 - 102), sense	Varroa actin mRNA qRT-PCR
**Amel-qbACT-F**	AGGAATGGAAGCTTGCGGTA	NM_001185146, (919 - 938), sense	Honey bee actin mRNA RT-qPCR
**Amel-qbACT-R**	AATTTTCATGGTGGATGGTGC	NM_001185146, (1099 - 1079), complement	Honey bee actin mRNA RT-qPCR
**DWV900F**	GTTAATGTCTCATAGCCCAGACG	AY292384, (899 - 921), sense	RT-PCR, 1.2 kbp fragment, DWV strain identification
**DWV2050Rev**	CATATAGCATCAGAATTAGCCTC	AY292384, (2071 - 2049), complement	RT-PCR, 1.2 kbp fragment, DWV strain identification
**APO3Q-F1**	GCGAAATCTCTGAGCCAAC	NM_001114198, (473 - 491), sense	Honey bee Apo3 mRNA RT-qPCR
**APO3Q-R1**	GAGTTGCGGCAGTTTGAAG	NM_001114198 (599 - 581), complement	Honey bee Apo3 mRNA RT-qPCR
**Tag1-DWV-For**	CTTGGTTAGCTGTGTTGCAGTTGCT GTAGTCAAGCGGTTACTTGAG	AY292384, (4909 - 4934), sense	Negative-strand DWV RNA detection (Assay 1), RT
**DWV-1-rev**	GGAGCTTCTGGAACGGCAGAT	AY292384, (5008 - 4988), complement	Negative-strand DWV RNA detection (Assay 1), qPCR
**Tag1**	CTTGGTTAGCTGTGTTGCAGTTG	not applicable	Negative-strand DWV RNA detection (Assay 1), qPCR
**Tag2-DWV-2-For**	AGCCTGCGCACGTGGcgaaaccaacttct gaggaa	AY292384, (7330 - 7349), sense	Negative-strand DWV RNA detection (Assay 2), RT
**DWV-2-rev**	GTGTTGATCCCTGAGGCTTA	AY292384, (7503 - 7484), complement	Negative-strand DWV RNA detection (Assay 2), qPCR
**Tag2**	AGCCTGCGCACGTGG	not applicable	Negative-strand DWV RNA detection (Assay 2), qPCR

Supplementary Table 2. Detection of honey bee mRNA in *Varroa* mites by RT-qPCR. Ct values for honey bee actin (BeeBase gene identifier GB44311) and Apolipophorin-III-like, Apo3 (BeeBase gene identifier GB55452) mRNAs, *Varroa destructor* actin (GenBank Accession XR_002672520) mRNA, and DWV genomic RNA in pooled *Varroa* mite and control honey bee pupae.

Pooled samples	DWV RNA	Varroa actin mRNA	Honey bee actin mRNA	Honey bee Apo3 mRNA
***Varroa* mites**				
**V-Pho$_1$**	25.8	19.7	26.8	36.6
**V0-PBS$_1$**	nd	21.9	31.0	nd
**V0-DWV$_1$**	14.7	22.8	36.4	41.2
**V4-PBS$_1$**	25.1	24.4	34.2	nd
**V4-DWV$_1$**	21.5	22.8	33.5	37.9
**Honey bee pupae**				
**P0-DWV$_1$**	11.0	nd	17.3	23.6
**P4-DWV$_1$**	18.0	nd	24.3	31.3
**Water control**	nd	nd	nd	nd

nd - not detected

Supplementary Table 3. Detection and quantification of honey bee mRNAs in RNAseq libraries from phoretic *Varroa* mites following starvation or feeding. Numbers of NGS reads corresponding a set of *Varroa* and honey bee mRNAs as well as DWV genomic RNA were determined in *Varroa destructor* RNAseq libraries (Egekwu & Cook, submitted).

**Varroa RNA-seq library ID**		**Var_C333_VaD_WaS_12H**	**Var_C3_VaD_WaF_15H**	**Var_C3_VaD_WpF_15H**
***Varroa* mite treatment**		Phoretic mites starved for 12 hours	Phoretic mites fed on adult honey bees for 15 hours	Phoretic mites fed on honey bee pupae for 15 hours
**Total number of reads in the library**		59560566	49673302	65165306
**DWV reads**		38870677	18964968	24572362
**Reads numbers ratio, honey bee beta-actin mRNA to *Varroa* beta-actin**		0.017	0.039	0.034
**Reads numbers ratio, total honey bee mRNAs to *Varroa* beta-actin mRNA**		2.540	24.152	19.066
***Apis mellifera* mRNA**	***A. mellifera beta-* actin (GB44311) NM_001185146**	626	674	836
	***A. mellifera* myosin heavy chain (GB51653)**	1428	4094	5231
	***A. mellifera* apolipophorin-III-like protein (GB55452)**	1	240	220
**Total *A. mellifera* mRNAs reads**		92604	414034	470256
***Varroa destructor* mRNA**	**XR_002672520, *V. destructor* beta actin**	36462	17143	24665
	***V. destructor* myosin heavy chain**	2246	3318	4859
	***V. destructor* vitellogenin 1 JQ974976**	1901	773	1289

Supplementary Table 4. Detection of honey bee mRNAs in RNAseq libraries from different *Varroa* mite developmental stages. Numbers of NGS reads corresponding a set of *Varroa* and honey bee mRNAs were determined in *Varroa destructor* RNAseq libraries[23] (NCBI BioProject PRJNA392105).

Varroa destructor RNA-seq library, accession number, mite description		SRX2960636 Emerging mites (collected from P8 to P9 brood cells	SRX29606371 Young mites (collected from P8 to P9 brood cells)	SRX2960647 Young mites (collected from P8 to P9 brood cells)	SRX2960634 Laying mites (collected from sealed brood cells containing pre-pupae)	SRX2960649 Phoretic mites (collected on adult bees)	SRX2960670 Phoretic mites artificially reared in cages with adult bees	SRX2960669 Phoretic mites (collected on adult bees)	SRX2960632 Arresting mites (collected in unsealed L5 brood cells)
Total number of reads in the library		26531852	27083570	33433690	26670037	17467280	29446920	32563456	28709594
Apis mellifera mRNAs	NM_001185146 *A. mellifera* actin related protein 1 (Arp1)	140	473	683	174	93	206	88	87
	XM_02644216 *A. mellifera* myosin heavy chain	122	758	355	36	56	92	57	14
	NM_001114198 *A. mellifera* apolipophorin-III-like protein	3	51	77	11	1	0	0	2
Varroa destructor mRNAs	XR_002672520, *V. destructor* actin-like protein	>20000	>20000	>20000	8606	17838	>20000	13931	9678
	XM_022800959 *V. destructor* myosin heavy chain	2314	2523	4080	1847	1494	2920	1017	1061
	JQ974976 *V. destructor* vitellogenin 1	1649	524	1182	>20000	848	1446	756	3405

Supplementary Figure S1. Alignments of consensus DWV RNA nucleotide sequences from honey bees and mites in Experiment 2: "*Varroa*-mediated DWV transmission experiment". RT-PCR fragments corresponding to the DWV LP region were amplified using RNA extracted from bees and mites pooled according to their treatment (Experiment 2, Figs. 2 and 4). **(a, b)** Complete identity between the DWV sequences amplified from honey bee pupae P0-DWV$_2$, P1-DWV$_2$, and the cloned isolate DWV-304. **(c, d)** Complete nucleotide identity between the sequenced terminal portions of the RT-PCR fragment amplified from V0-DWV$_2$ mites, the sequences from honey bee pupae P0-DWV$_2$, P1-DWV$_2$, and the cloned isolate DWV-304. Due to high polymorphism in the mite DWV load only the terminal portions of V0-DWV$_2$ were sequenced. Cloned DWV isolate DWV-304 (GenBank accession number MG831200, isolated in the USA in 2015) was used for pupae P0-DWV$_2$ injection. The type DWV isolate DWV-PA isolated the USA in 2006 (GenBank Accession number AY292384), was included with alignments **(b)** and **(c)** to show distribution of polymorphic sites in the LP region of DWV RNA.

(a)

```
DWV-304                 agacatcatattttattttaatgctgtctttattgctgatttattttgctgtttttattt        1020
Pupae-P0-DWV-2          -------------TATTTTAATGCTGTCTTTATTGCTGATTTATTTTGCTGTTTTTATTT        47
Pupae-P1-DWV—Ext-2      ------CATATTTTATTTTAATGCTGTCTTTATTGCTGATTTATTTTGCTGTTTTTATTT        54
                                     ***********************************************

DWV-304                 gctattttatatttgctaattttcattattgcgaaatatattacattgctatttttatta        1080
Pupae-P0-DWV-2          GCTATTTTATATTTGCTAATTTTCATTATTGCGAAATATATTACATTGCTATTTTTATTA        107
Pupae-P1-DWV—Ext-2      GCTATTTTATATTTGCTAATTTTCATTATTGCGAAATATATTACATTGCTATTTTTATTA        114
                        ************************************************************

DWV-304                 tatacgctagattcaatttttatttttcctatattttcaatttaattttgattttgaaggt        1140
Pupae-P0-DWV-2          TATACGCTAGATTCAATTTTATTTTTCCTATATTTTCAATTTAATTTTGATTTTGAAGGT        167
Pupae-P1-DWV—Ext-2      TATACGCTAGATTCAATTTTATTTTTCCTATATTTTCAATTTAATTTTGATTTTGAAGGT        174
                        ************************************************************

DWV-304                 aaatatatataattaattattaaaaatggccttcagttgtggaactctctcctactctgc        1200
Pupae-P0-DWV-2          AAATATATATAATTAATTATTAAAAATGGCCTTCAGTTGTGGAACTCTCTCCTACTCTGC        227
Pupae-P1-DWV—Ext-2      AAATATATATAATTAATTATTAAAAATGGCCTTCAGTTGTGGAACTCTCTCCTACTCTGC        234
                        ************************************************************

DWV-304                 tgtcgcccaagctccgtccgttgcccatgcacctcgtacatgggaagttgatgaagccag        1260
Pupae-P0-DWV-2          TGTCGCCCAAGCTCCGTCCGTTGCCCATGCACCTCGTACATGGGAAGTTGATGAAGCCAG        287
Pupae-P1-DWV—Ext-2      TGTCGCCCAAGCTCCGTCCGTTGCCCATGCACCTCGTACATGGGAAGTTGATGAAGCCAG        294
                        ************************************************************

DWV-304                 gcggcgccgagtcatcaaacgtttggcgctggagcaagaacgtattcgcaacgttcttga        1320
Pupae-P0-DWV-2          GCGGCGCCGAGTCATCAAACGTTTGGCGCTGGAGCAAGAACGTATTCGCAACGTTCTTGA        347
Pupae-P1-DWV—Ext-2      GCGGCGCCGAGTCATCAAACGTTTGGCGCTGGAGCAAGAACGTATTCGCAACGTTCTTGA        354
                        ************************************************************

DWV-304                 cgccggcgtctatgaccaggcgacatgggaacaggaggacgcgcgcgataatgagttcct        1380
Pupae-P0-DWV-2          CGCCGGCGTCTATGACCAGGCGACATGGGAACAGGAGGACGCGCGCGATAATGAGTTCCT        407
Pupae-P1-DWV—Ext-2      CGCCGGCGTCTATGACCAGGCGACATGGGAACAGGAGGACGCGCGCGATAATGAGTTCCT        414
                        ************************************************************

DWV-304                 aacggaacaattaaacaatttatatactatttattcgatcgccgaacgttgcacgcgtcg        1440
Pupae-P0-DWV-2          AACGGAACAATTAAACAATTTATATACTATTTATTCGATCGCCGAACGTTGCACGCGTCG        467
Pupae-P1-DWV—Ext-2      AACGGAACAATTAAACAATTTATATACTATTTATTCGATCGCCGAACGTTGCACGCGTCG        474
                        ************************************************************

DWV-304                 acctattaaagagcactctcctatatcagtttcgaataggtttgctccattggaatccct        1500
Pupae-P0-DWV-2          ACCTATTAAAGAGCACTCTCCTATATCAGTTTCGAATAGGTTTGCTCCATTGGAATCCCT        527
Pupae-P1-DWV—Ext-2      ACCTATTAAAGAGCACTCTCCTATATCAGTTTCGAATAGGTTTGCTCCATTGGAATCCCT        534
                        ************************************************************

DWV-304                 caaagtcgaggtcggtcaagaagcaggcgaatgtatatttaagaaacctaaatacacgcg        1560
Pupae-P0-DWV-2          CAAAGTCGAGGTCGGTCAAGAAGCAGGCGAATGTATATTTAAGAAACCTAAATACACGCG        587
Pupae-P1-DWV—Ext-2      CAAAGTCGAGGTCGGTCAAGAAGCAGGCGAATGTATATTTAAGAAACCTAAATACACGCG        594
                        ************************************************************
```

```
DWV-304                   cgtttgcaagaaagtgaagcgtgttgcaacccgcttcgttcgtgaaaaagttgttcgtcc      1620
Pupae-P0-DWV-2            CGTTTGCAAGAAAGTGAAGCGTGTTGCAACCCGCTTCGTTCGTGAAAAAGTTGTTCGTCC      647
Pupae-P1-DWV—Ext-2        CGTTTGCAAGAAAGTGAAGCGTGTTGCAACCCGCTTCGTTCGTGAAAAAGTTGTTCGTCC      654
                          ************************************************************

DWV-304                   tatgtgttctagatctcctatgctattatttaagcttaagaaaattatttatgatttgca      1680
Pupae-P0-DWV-2            TATGTGTTCTAGATCTCCTATGCTATTATTTAAGCTTAAGAAAATTATTTATGATTTGCA      707
Pupae-P1-DWV—Ext-2        TATGTGTTCTAGATCTCCTATGCTATTATTTAAGCTTAAGAAAATTATTTATGATTTGCA      714
                          ************************************************************

DWV-304                   cttatatagattaagaaaacagattaggatttgagacgtcaaaaacagcgcgagtatga      1740
Pupae-P0-DWV-2            CTTATATAGATTAAGAAAACAGATTAGGATTTGAGACGTCAAAAACAGCGCGAGTATGA      767
Pupae-P1-DWV—Ext-2        CTTATATAGATTAAGAAAACAGATTAGGATTTGAGACGTCAAAAACAGCGCGAGTATGA      774
                          ************************************************************

DWV-304                   gttagagtgtgtcactaatctgttacaattatcgaatccggtgcaggcaaaaccagagat      1800
Pupae-P0-DWV-2            GTTAGAGTGTGTCACTAATCTGTTACAATTATCGAATCCGGTGCAGGCAAAACCAGAGAT      827
Pupae-P1-DWV—Ext-2        GTTAGAGTGTGTCACTAATCTGTTACAATTATCGAATCCGGTGCAGGCAAAACCAGAGAT      834
                          ************************************************************

DWV-304                   ggataaccctaatccaggacctgatggcgagggtgaagttgaattagaaaaggatagtaa      1860
Pupae-P0-DWV-2            GGATAACCCTAATCCAGGACCTGATGGCGAGGGTGAAGTTGAATTAGAAAAGGATAGTAA      887
Pupae-P1-DWV—Ext-2        GGATAACCCTAATCCAGGACCTGATGGCGAGGGTGAAGTTGAATTAGAAAAGGATAGTAA      894
                          ************************************************************

DWV-304                   tgttgttttaacaactcagcgagatcctagtacatctattccagcgccggtgagcgtaaa      1920
Pupae-P0-DWV-2            TGTTGTTTTAACAACTCAGCGAGATCCTAGTACATCTATTCCAGCGCCGGTGAGCGTAAA      947
Pupae-P1-DWV—Ext-2        TGTTGTTTTAACAACTCAGCGAGATCCTAGTACATCTATTCCAGCGCCGGTGAGCGTAAA      954
                          ************************************************************

DWV-304                   atggagtagatggactagtaatgatgtagtagatgattacgccacaatcacatctcgatg      1980
Pupae-P0-DWV-2            ATGGAGTAGATGGACTAGTAATGATGTAGTAGATGATTACGCCACAATCACATCTCGATG      1007
Pupae-P1-DWV—Ext-2        ATGGAGTAGATGGACTAGTAATGATGTAGTAGATGATTACGCCACAATCACATCTCGATG      1014
                          ************************************************************

DWV-304                   gtatcagattgctgaatttgtttggtcgaaggatgatccatttgataaggagttagcacg      2040
Pupae-P0-DWV-2            GTATCAGATTGCTGAATTTGTTTGGTCGAAGGATGATCCATTTGATAAGGAGTTAGCACG      1067
Pupae-P1-DWV—Ext-2        GTATCAGATTGCTGAATTTGTTTGGTCGAAGGATGATCCATTTGATAAGGAGTTAGCACG      1074
                          ************************************************************
```

(b)

```
DWV-PA                    AGACATCATATTTTATTTTAATGCTGTCTTTATTGCTGATTTATCTTGCTGTTTTTATTT      994
DWV-304                   agacatcatattttattttaatgctgtctttattgctgatttattttgctgtttttattt      1020
Pupae-P0-DWV-2            -------------TATTTTAATGCTGTCTTTATTGCTGATTTATTTTGCTGTTTTTATTT      47
Pupae-P1-DWV—Ext-2        ------CATATTTTATTTTAATGCTGTCTTTATTGCTGATTTATTTTGCTGTTTTTATTT      54
                                       ******************************* ***************

DWV-PA                    GCTATTTTATATTTGCTAATTTTCATTATTGCGAAATATATTACATTGCTATTTTTATTA      1054
DWV-304                   gctattttatatttgctaattttcattattgcgaaatatattacattgctatttttatta      1080
Pupae-P0-DWV-2            GCTATTTTATATTTGCTAATTTTCATTATTGCGAAATATATTACATTGCTATTTTTATTA      107
Pupae-P1-DWV—Ext-2        GCTATTTTATATTTGCTAATTTTCATTATTGCGAAATATATTACATTGCTATTTTTATTA      114
                          ************************************************************

DWV-PA                    TATACGCTAGATTCAATTTTATTTTTTCTATATTTTCAATTTAATTTTGATTTCGAAGGT      1114
DWV-304                   tatacgctagattcaattttatttttcctatattttcaatttaattttgattttgaaggt      1140
Pupae-P0-DWV-2            TATACGCTAGATTCAATTTTATTTTTCCTATATTTTCAATTTAATTTTGATTTTGAAGGT      167
Pupae-P1-DWV—Ext-2        TATACGCTAGATTCAATTTTATTTTTCCTATATTTTCAATTTAATTTTGATTTTGAAGGT      174
                          ************************** ************************** ******

DWV-PA                    AAATATATATAATTAATTATTAAAAATGGCCTTTAGTTGTGGAACTCTTTCTTACTCTGC      1174
DWV-304                   aaatatatataattaattattaaaaatggccttcagttgtggaactctctcctactctgc      1200
Pupae-P0-DWV-2            AAATATATATAATTAATTATTAAAAATGGCCTTCAGTTGTGGAACTCTCTCCTACTCTGC      227
Pupae-P1-DWV—Ext-2        AAATATATATAATTAATTATTAAAAATGGCCTTCAGTTGTGGAACTCTCTCCTACTCTGC      234
                          ********************************* ************** ** ********

DWV-PA                    CGTCGCCCAAGCTCCGTCTGTCGCCTATGCACCTCGTACATGGGAAGTTGATGAAGCTAG      1234
DWV-304                   tgtcgcccaagctccgtccgttgcccatgcacctcgtacatgggaagttgatgaagccag      1260
Pupae-P0-DWV-2            TGTCGCCCAAGCTCCGTCCGTTGCCCATGCACCTCGTACATGGGAAGTTGATGAAGCCAG      287
Pupae-P1-DWV—Ext-2        TGTCGCCCAAGCTCCGTCCGTTGCCCATGCACCTCGTACATGGGAAGTTGATGAAGCCAG      294
                           ***************** ** *** ******************************* **
```

```
DWV-PA                    GCGGCGCCGAGTCATTAAACGTTTGGCGCTGGAGCAAGAACGTATTCGTAACGTTCTTGA        1294
DWV-304                   gcggcgccgagtcatcaaacgtttggcgctggagcaagaacgtattcgcaacgttcttga        1320
Pupae-P0-DWV-2            GCGGCGCCGAGTCATCAAACGTTTGGCGCTGGAGCAAGAACGTATTCGCAACGTTCTTGA        347
Pupae-P1-DWV-Ext-2        GCGGCGCCGAGTCATCAAACGTTTGGCGCTGGAGCAAGAACGTATTCGCAACGTTCTTGA        354
                          *************** ******************************** ***********

DWV-PA                    CGTTGGCGTCTATGACCAGGCGACATGGGAACAGGAGGACGCGCGCGATAATGAGTTCCT        1354
DWV-304                   cgccggcgtctatgaccaggcgacatgggaacaggaggacgcgcgcgataatgagttcct        1380
Pupae-P0-DWV-2            CGCCGGCGTCTATGACCAGGCGACATGGGAACAGGAGGACGCGCGCGATAATGAGTTCCT        407
Pupae-P1-DWV-Ext-2        CGCCGGCGTCTATGACCAGGCGACATGGGAACAGGAGGACGCGCGCGATAATGAGTTCCT        414
                          **  ********************************************************

DWV-PA                    AACGGAACAATTAAACAATTTATATACTATTTATTCGATCGCTGAACGTTGTACGCGTCG        1414
DWV-304                   aacggaacaattaaacaatttatatactatttattcgatcgccgaacgttgcacgcgtcg        1440
Pupae-P0-DWV-2            AACGGAACAATTAAACAATTTATATACTATTTATTCGATCGCCGAACGTTGCACGCGTCG        467
Pupae-P1-DWV-Ext-2        AACGGAACAATTAAACAATTTATATACTATTTATTCGATCGCCGAACGTTGCACGCGTCG        474
                          ****************************************** ******** ********

DWV-PA                    GCCTATCAAAGAGTACTCTCCTATATCAGTTTCGAATAGGTTTGCTCCACTGGAATCCCT        1474
DWV-304                   acctattaaagagcactctcctatatcagtttcgaataggtttgctccattggaatccct        1500
Pupae-P0-DWV-2            ACCTATTAAAGAGCACTCTCCTATATCAGTTTCGAATAGGTTTGCTCCATTGGAATCCCT        527
Pupae-P1-DWV-Ext-2        ACCTATTAAAGAGCACTCTCCTATATCAGTTTCGAATAGGTTTGCTCCATTGGAATCCCT        534
                           ***** ****** *********************************** **********

DWV-PA                    CAAGGTCGAGGTCGGTCAAGAAGCAGGCGAATGTATATTTAAGAAACCTAAATATACGCG        1534
DWV-304                   caaagtcgaggtcggtcaagaagcaggcgaatgtatatttaagaaacctaaatacacgcg        1560
Pupae-P0-DWV-2            CAAAGTCGAGGTCGGTCAAGAAGCAGGCGAATGTATATTTAAGAAACCTAAATACACGCG        587
Pupae-P1-DWV-Ext-2        CAAAGTCGAGGTCGGTCAAGAAGCAGGCGAATGTATATTTAAGAAACCTAAATACACGCG        594
                          *** ************************************************** *****

DWV-PA                    CGTTTGCAAGAAAGTGAAGCGTGTTGCAACTCGCTTCGTTCGTGAAAAAGTTGTTCGTCC        1594
DWV-304                   cgtttgcaagaaagtgaagcgtgttgcaacccgcttcgttcgtgaaaaagttgttcgtcc        1620
Pupae-P0-DWV-2            CGTTTGCAAGAAAGTGAAGCGTGTTGCAACCCGCTTCGTTCGTGAAAAAGTTGTTCGTCC        647
Pupae-P1-DWV-Ext-2        CGTTTGCAAGAAAGTGAAGCGTGTTGCAACCCGCTTCGTTCGTGAAAAAGTTGTTCGTCC        654
                          ****************************** *****************************

DWV-PA                    TATGTGTTCTAGATCCCCTATGCTATTATTTAAGCTTAAGAAAATTATTTATGATTTGCA        1654
DWV-304                   tatgtgttctagatctcctatgctattatttaagcttaagaaaattatttatgatttgca        1680
Pupae-P0-DWV-2            TATGTGTTCTAGATCTCCTATGCTATTATTTAAGCTTAAGAAAATTATTTATGATTTGCA        707
Pupae-P1-DWV-Ext-2        TATGTGTTCTAGATCTCCTATGCTATTATTTAAGCTTAAGAAAATTATTTATGATTTGCA        714
                          *************** ********************************************

DWV-PA                    CTTATATAGATTAAGAAAACAGATTAGGATGTTGAGACGTCAAAAACAGCGCGATTACGA        1714
DWV-304                   cttatatagattaagaaaacagattaggattttgagacgtcaaaaacagcgcgagtatga        1740
Pupae-P0-DWV-2            CTTATATAGATTAAGAAAACAGATTAGGATTTTGAGACGTCAAAAACAGCGCGAGTATGA        767
Pupae-P1-DWV-Ext-2        CTTATATAGATTAAGAAAACAGATTAGGATTTTGAGACGTCAAAAACAGCGCGAGTATGA        774
                          ****************************** *********************** ** **

DWV-PA                    GTTAGAGTGTGTCACTAATCTGTTACAATTATCGAATCCGGTGCAGGCAAAACCAGAGAT        1774
DWV-304                   gttagagtgtgtcactaatctgttacaattatcgaatccggtgcaggcaaaaccagagat        1800
Pupae-P0-DWV-2            GTTAGAGTGTGTCACTAATCTGTTACAATTATCGAATCCGGTGCAGGCAAAACCAGAGAT        827
Pupae-P1-DWV-Ext-2        GTTAGAGTGTGTCACTAATCTGTTACAATTATCGAATCCGGTGCAGGCAAAACCAGAGAT        834
                          ************************************************************

DWV-PA                    GGATAACCCTAATCCAGGACCTGATGGCGAGGGTGAAGTTGAATTAGAAAAGGATAGTAA        1834
DWV-304                   ggataaccctaatccaggacctgatggcgagggtgaagttgaattagaaaaggatagtaa        1860
Pupae-P0-DWV-2            GGATAACCCTAATCCAGGACCTGATGGCGAGGGTGAAGTTGAATTAGAAAAGGATAGTAA        887
Pupae-P1-DWV-Ext-2        GGATAACCCTAATCCAGGACCTGATGGCGAGGGTGAAGTTGAATTAGAAAAGGATAGTAA        894
                          ************************************************************

DWV-PA                    TGTTGTTTTAACAACTCAGCGAGATCCTAGTACATCTATTCCAGCGCCGGTGAGCGTAAA        1894
DWV-304                   tgttgttttaacaactcagcgagatcctagtacatctattccagcgccggtgagcgtaaa        1920
Pupae-P0-DWV-2            TGTTGTTTTAACAACTCAGCGAGATCCTAGTACATCTATTCCAGCGCCGGTGAGCGTAAA        947
Pupae-P1-DWV-Ext-2        TGTTGTTTTAACAACTCAGCGAGATCCTAGTACATCTATTCCAGCGCCGGTGAGCGTAAA        954
                          ************************************************************

DWV-PA                    ATGGAGTAGATGGACTAGTAATGATGTAGTAGATGATTACGCCACAATCACATCTCGATG        1954
DWV-304                   atggagtagatggactagtaatgatgtagtagatgattacgccacaatcacatctcgatg        1980
Pupae-P0-DWV-2            ATGGAGTAGATGGACTAGTAATGATGTAGTAGATGATTACGCCACAATCACATCTCGATG        1007
Pupae-P1-DWV-Ext-2        ATGGAGTAGATGGACTAGTAATGATGTAGTAGATGATTACGCCACAATCACATCTCGATG        1014
                          ************************************************************


DWV-PA                    GTATCAGATTGCTGAATTTGTTTGGTCGAAGGATGATCCATTTGATAAGGAGTTAGCACG        2014
DWV-304                   gtatcagattgctgaatttgtttggtcgaaggatgatccatttgataaggagttagcacg        2040
Pupae-P0-DWV-2            GTATCAGATTGCTGAATTTGTTTGGTCGAAGGATGATCCATTTGATAAGGAGTTAGCACG        1067
Pupae-P1-DWV-Ext-2        GTATCAGATTGCTGAATTTGTTTGGTCGAAGGATGATCCATTTGATAAGGAGTTAGCACG        1074
                          ************************************************************
```

-

(c)

```
V0-DWV-2                  -------------TCATATTTTGCTGTCTTTATTGCTGATTTATTTTGCTGTTTTTATTT       55
DWV-304                   agacatcatattttattttaatgctgtctttattgctgatttattttgctgtttttattt       1020
Pupae-P0-DWV-2            -------------TATTTTAATGCTGTCTTTATTGCTGATTTATTTTGCTGTTTTTATTT       47
Pupae-P1-DWV—Ext-2        ------CATATTTTATTTTAATGCTGTCTTTATTGCTGATTTATTTTGCTGTTTTTATTT       54
                                       ***********************************************

V0-DWV-2                  GCTATTTTATATTTGCTAATTTTCATTATTGCGAAATATATTACATTGCTATTTTTATTA       115
DWV-304                   gctattttatatttgctaattttcattattgcgaaatatattacattgctatttttatta       1080
Pupae-P0-DWV-2            GCTATTTTATATTTGCTAATTTTCATTATTGCGAAATATATTACATTGCTATTTTTATTA       107
Pupae-P1-DWV—Ext-2        GCTATTTTATATTTGCTAATTTTCATTATTGCGAAATATATTACATTGCTATTTTTATTA       114
                          ************************************************************

V0-DWV-2                  TATACGCTAGATTCAATTTTATTTTTCCTATATTTTCAATTTAATTTTGATTTTGAAGGT       175
DWV-304                   tatacgctagattcaattttatttttcctatattttcaatttaattttgattttgaaggt       1140
Pupae-P0-DWV-2            TATACGCTAGATTCAATTTTATTTTTCCTATATTTTCAATTTAATTTTGATTTTGAAGGT       167
Pupae-P1-DWV—Ext-2        TATACGCTAGATTCAATTTTATTTTTCCTATATTTTCAATTTAATTTTGATTTTGAAGGT       174
                          ************************************************************

V0-DWV-2                  AAATATATATAATTAATTATTAAAAATGGCCTnnnnnnnnnnnnnnnnnnnnnnnnnnnn       235
DWV-304                   aaatatatataattaattattaaaaatggccttcagttgtggaactctctcctactctgc       1200
Pupae-P0-DWV-2            AAATATATATAATTAATTATTAAAAATGGCCTTCAGTTGTGGAACTCTCTCCTACTCTGC       227
Pupae-P1-DWV—Ext-2        AAATATATATAATTAATTATTAAAAATGGCCTTCAGTTGTGGAACTCTCTCCTACTCTGC       234
                          ********************************

V0-DWV-2                  nnnnnnnnnnnnnnnnnnnnnnnnnnnnnnnnnnnnnnnnnnnnnnnnnnnnnnnnnnnn       295
DWV-304                   tgtcgcccaagctccgtccgttgcccatgcacctcgtacatgggaagttgatgaagccag       1260
Pupae-P0-DWV-2            TGTCGCCCAAGCTCCGTCCGTTGCCCATGCACCTCGTACATGGGAAGTTGATGAAGCCAG       287
Pupae-P1-DWV—Ext-2        TGTCGCCCAAGCTCCGTCCGTTGCCCATGCACCTCGTACATGGGAAGTTGATGAAGCCAG       294

V0-DWV-2                  nnnnnnnnnnnnnnnnnnnnnnnnnnnnnnnnnnnnnnnnnnnnnnnnnnnnnnnnnnnn       355
DWV-304                   gcggcgccgagtcatcaaacgtttggcgctggagcaagaacgtattcgcaacgttcttga       1320
Pupae-P0-DWV-2            GCGGCGCCGAGTCATCAAACGTTTGGCGCTGGAGCAAGAACGTATTCGCAACGTTCTTGA       347
Pupae-P1-DWV—Ext-2        GCGGCGCCGAGTCATCAAACGTTTGGCGCTGGAGCAAGAACGTATTCGCAACGTTCTTGA       354

V0-DWV-2                  nnnnnnnnnnnnnnnnnnnnnnnnnnnnnnnnnnnnnnnnnnnnnnnnnnnnnnnnnnnn       415
DWV-304                   cgccggcgtctatgaccaggcgacatgggaacaggaggacgcgcgcgataatgagttcct       1380
Pupae-P0-DWV-2            CGCCGGCGTCTATGACCAGGCGACATGGGAACAGGAGGACGCGCGCGATAATGAGTTCCT       407
Pupae-P1-DWV—Ext-2        CGCCGGCGTCTATGACCAGGCGACATGGGAACAGGAGGACGCGCGCGATAATGAGTTCCT       414

V0-DWV-2                  nnnnnnnnnnnnnnnnnnnnnnnnnnnnnnnnnnnnnnnnnnnnnnnnnnnnnnnnnnnn       475
DWV-304                   aacggaacaattaaacaatttatatactatttattcgatcgccgaacgttgcacgcgtcg       1440
Pupae-P0-DWV-2            AACGGAACAATTAAACAATTTATATACTATTTATTCGATCGCCGAACGTTGCACGCGTCG       467
Pupae-P1-DWV—Ext-2        AACGGAACAATTAAACAATTTATATACTATTTATTCGATCGCCGAACGTTGCACGCGTCG       474

V0-DWV-2                  nnnnnnnnnnnnnnnnnnnnnnnnnnnnnnnnnnnnnnnnnnnnnnnnnnnnnnnnnnnn       535
DWV-304                   acctattaaagagcactctcctatatcagtttcgaataggtttgctccattggaatccct       1500
Pupae-P0-DWV-2            ACCTATTAAAGAGCACTCTCCTATATCAGTTTCGAATAGGTTTGCTCCATTGGAATCCCT       527
Pupae-P1-DWV—Ext-2        ACCTATTAAAGAGCACTCTCCTATATCAGTTTCGAATAGGTTTGCTCCATTGGAATCCCT       534

V0-DWV-2                  nnnnnnnnnnnnnnnnnnnnnnnnnnnnnnnnnnnnnnnnnnnnnnnnnnnnnnnnnnnn       594
DWV-304                   caaagtcgaggtcggtcaagaagcaggcgaatgtatatttaagaaacctaaatacacgcg       1560
Pupae-P0-DWV-2            CAAAGTCGAGGTCGGTCAAGAAGCAGGCGAATGTATATTTAAGAAACCTAAATACACGCG       587
Pupae-P1-DWV—Ext-2        CAAAGTCGAGGTCGGTCAAGAAGCAGGCGAATGTATATTTAAGAAACCTAAATACACGCG       594

V0-DWV-2                  nnnnnnnnnnnnnnnnnnnnnnnnnnnnnnnnnnnnnnnnnnnnnnnnnnnnnnnnnnnn       654
DWV-304                   cgtttgcaagaaagtgaagcgtgttgcaacccgcttcgttcgtgaaaaagttgttcgtcc       1620
Pupae-P0-DWV-2            CGTTTGCAAGAAAGTGAAGCGTGTTGCAACCCGCTTCGTTCGTGAAAAAGTTGTTCGTCC       647
Pupae-P1-DWV—Ext-2        CGTTTGCAAGAAAGTGAAGCGTGTTGCAACCCGCTTCGTTCGTGAAAAAGTTGTTCGTCC       654

V0-DWV-2                  nnnnnnnnnnnnnnnnnnnnnnnnnnnnnnnnnnnnnnnnnnnnnnnnnnnnnnnnnnnn       714
DWV-304                   tatgtgttctagatctcctatgctattatttaagcttaagaaaattatttatgatttgca       1680
Pupae-P0-DWV-2            TATGTGTTCTAGATCTCCTATGCTATTATTTAAGCTTAAGAAAATTATTTATGATTTGCA       707
Pupae-P1-DWV—Ext-2        TATGTGTTCTAGATCTCCTATGCTATTATTTAAGCTTAAGAAAATTATTTATGATTTGCA       714

V0-DWV-2                  nnnnnnnnnnnnnnnnnnnnnnnnnnnnnnnnnnnnnnnnnnnnnnnnnnnnnnnnnnnn       774
DWV-304                   cttatatagattaagaaaacagattaggattttgagacgtcaaaaacagcgcgagtatga       1740
Pupae-P0-DWV-2            CTTATATAGATTAAGAAAACAGATTAGGATTTTGAGACGTCAAAAACAGCGCGAGTATGA       767
Pupae-P1-DWV—Ext-2        CTTATATAGATTAAGAAAACAGATTAGGATTTTGAGACGTCAAAAACAGCGCGAGTATGA       774
```

```
V0-DWV-2                  nnnnnnnnnnnnnnnTAATCTGTTACAATTATCGAATCCGGTGCAGGCAAAACCAGAGAT          834
DWV-304                   gttagagtgtgtcactaatctgttacaattatcgaatccggtgcaggcaaaaccagagat          1800
Pupae-P0-DWV-2            GTTAGAGTGTGTCACTAATCTGTTACAATTATCGAATCCGGTGCAGGCAAAACCAGAGAT          827
Pupae-P1-DWV—Ext-2        GTTAGAGTGTGTCACTAATCTGTTACAATTATCGAATCCGGTGCAGGCAAAACCAGAGAT          834
                                         *********************************************

V0-DWV-2                  GGATAACCCTAATCCAGGACCTGATGGCGAGGGTGAAGTTGAATTAGAAAAGGATAGTAA          894
DWV-304                   ggataaccctaatccaggacctgatggcgagggtgaagttgaattagaaaaggatagtaa          1860
Pupae-P0-DWV-2            GGATAACCCTAATCCAGGACCTGATGGCGAGGGTGAAGTTGAATTAGAAAAGGATAGTAA          887
Pupae-P1-DWV—Ext-2        GGATAACCCTAATCCAGGACCTGATGGCGAGGGTGAAGTTGAATTAGAAAAGGATAGTAA          894
                          ************************************************************

V0-DWV-2                  TGTTGTTTTAACAACTCAGCGAGATCCTAGTACATCTATTCCAGCGCCGGTGAGCGTAAA          954
DWV-304                   tgttgttttaacaactcagcgagatcctagtacatctattccagcgccggtgagcgtaaa          1920
Pupae-P0-DWV-2            TGTTGTTTTAACAACTCAGCGAGATCCTAGTACATCTATTCCAGCGCCGGTGAGCGTAAA          947
Pupae-P1-DWV—Ext-2        TGTTGTTTTAACAACTCAGCGAGATCCTAGTACATCTATTCCAGCGCCGGTGAGCGTAAA          954
                          ************************************************************

V0-DWV-2                  ATGGAGTAGATGGACTAGTAATGATGTAGTAGATGATTACGCCACAATCACATCTCGATG          1014
DWV-304                   atggagtagatggactagtaatgatgtagtagatgattacgccacaatcacatctcgatg          1980
Pupae-P0-DWV-2            ATGGAGTAGATGGACTAGTAATGATGTAGTAGATGATTACGCCACAATCACATCTCGATG          1007
Pupae-P1-DWV—Ext-2        ATGGAGTAGATGGACTAGTAATGATGTAGTAGATGATTACGCCACAATCACATCTCGATG          1014
                          ************************************************************

V0-DWV-2                  GTATCAGATTGCTGAATTTGTTTGGTCGAAGGATGATCCATTTGATAAGGAGTTAGCACG          1074
DWV-304                   gtatcagattgctgaatttgtttggtcgaaggatgatccatttgataaggagttagcacg          2040
Pupae-P0-DWV-2            GTATCAGATTGCTGAATTTGTTTGGTCGAAGGATGATCCATTTGATAAGGAGTTAGCACG          1067
Pupae-P1-DWV—Ext-2        GTATCAGATTGCTGAATTTGTTTGGTCGAAGGATGATCCATTTGATAAGGAGTTAGCACG          1074
                          ************************************************************
```

(d)

```
V0-DWV-2                  -----TCATATTTTATTTTAATGCTGTCTTTATTGCTGATTTATTTTGCTGTTTTTATTT          55
DWV-PA                    AGACATCATATTTTATTTTAATGCTGTCTTTATTGCTGATTTATCTTGCTGTTTTTATTT          994
DWV-304                   agacatcatattttattttaatgctgtctttattgctgatttattttgctgtttttattt          1020
Pupae-P0-DWV-2            -------------TATTTTAATGCTGTCTTTATTGCTGATTTATTTTGCTGTTTTTATTT          47
Pupae-P1-DWV—Ext-2        ------CATATTTTATTTTAATGCTGTCTTTATTGCTGATTTATTTTGCTGTTTTTATTT          54
                                       ****************************** ****************

V0-DWV-2                  GCTATTTTATATTTGCTAATTTTCATTATTGCGAAATATATTACATTGCTATTTTTATTA          115
DWV-PA                    GCTATTTTATATTTGCTAATTTTCATTATTGCGAAATATATTACATTGCTATTTTTATTA          1054
DWV-304                   gctattttatatttgctaattttcattattgcgaaatatattacattgctatttttatta          1080
Pupae-P0-DWV-2            GCTATTTTATATTTGCTAATTTTCATTATTGCGAAATATATTACATTGCTATTTTTATTA          107
Pupae-P1-DWV—Ext-2        GCTATTTTATATTTGCTAATTTTCATTATTGCGAAATATATTACATTGCTATTTTTATTA          114
                          ************************************************************

V0-DWV-2                  TATACGCTAGATTCAATTTTATTTTTCCTATATTTTCAATTTAATTTTGATTTTGAAGGT          175
DWV-PA                    TATACGCTAGATTCAATTTTATTTTTTCTATATTTTCAATTTAATTTTGATTTCGAAGGT          1114
DWV-304                   tatacgctagattcaattttatttttcctatattttcaatttaattttgattttgaaggt          1140
Pupae-P0-DWV-2            TATACGCTAGATTCAATTTTATTTTTCCTATATTTTCAATTTAATTTTGATTTTGAAGGT          167
Pupae-P1-DWV—Ext-2        TATACGCTAGATTCAATTTTATTTTTCCTATATTTTCAATTTAATTTTGATTTTGAAGGT          174
                          ************************** ************************** ******

V0-DWV-2                  AAATATATATAATTAATTATTAAAAATGGCCTnnnnnnnnnnnnnnnnnnnnnnnnnnnn          235
DWV-PA                    AAATATATATAATTAATTATTAAAAATGGCCTTTAGTTGTGGAACTCTTTCTTACTCTGC          1174
DWV-304                   aaatatatataattaattattaaaaatggccttcagttgtggaactctctcctactctgc          1200
Pupae-P0-DWV-2            AAATATATATAATTAATTATTAAAAATGGCCTTCAGTTGTGGAACTCTCTCCTACTCTGC          227
Pupae-P1-DWV—Ext-2        AAATATATATAATTAATTATTAAAAATGGCCTTCAGTTGTGGAACTCTCTCCTACTCTGC          234
                          ********************************

V0-DWV-2                  nnnnnnnnnnnnnnnnnnnnnnnnnnnnnnnnnnnnnnnnnnnnnnnnnnnnnnnnnnnn          295
DWV-PA                    CGTCGCCCAAGCTCCGTCTGTCGCCTATGCACCTCGTACATGGGAAGTTGATGAAGCTAG          1234
DWV-304                   tgtcgcccaagctccgtccgttgcccatgcacctcgtacatgggaagttgatgaagccag          1260
Pupae-P0-DWV-2            TGTCGCCCAAGCTCCGTCCGTTGCCCATGCACCTCGTACATGGGAAGTTGATGAAGCCAG          287
Pupae-P1-DWV—Ext-2        TGTCGCCCAAGCTCCGTCCGTTGCCCATGCACCTCGTACATGGGAAGTTGATGAAGCCAG          294

V0-DWV-2                  nnnnnnnnnnnnnnnnnnnnnnnnnnnnnnnnnnnnnnnnnnnnnnnnnnnnnnnnnnnn          355
DWV-PA                    GCGGCGCCGAGTCATTAAACGTTTGGCGCTGGAGCAAGAACGTATTCGTAACGTTCTTGA          1294
DWV-304                   gcggcgccgagtcatcaaacgtttggcgctggagcaagaacgtattcgcaacgttcttga          1320
Pupae-P0-DWV-2            GCGGCGCCGAGTCATCAAACGTTTGGCGCTGGAGCAAGAACGTATTCGCAACGTTCTTGA          347
Pupae-P1-DWV—Ext-2        GCGGCGCCGAGTCATCAAACGTTTGGCGCTGGAGCAAGAACGTATTCGCAACGTTCTTGA          354

V0-DWV-2                  nnnnnnnnnnnnnnnnnnnnnnnnnnnnnnnnnnnnnnnnnnnnnnnnnnnnnnnnnnnn          415
DWV-PA                    CGTTGGCGTCTATGACCAGGCGACATGGGAACAGGAGGACGCGCGCGATAATGAGTTCCT          1354
DWV-304                   cgccggcgtctatgaccaggcgacatgggaacaggaggacgcgcgcgataatgagttcct          1380
Pupae-P0-DWV-2            CGCCGGCGTCTATGACCAGGCGACATGGGAACAGGAGGACGCGCGCGATAATGAGTTCCT          407
Pupae-P1-DWV—Ext-2        CGCCGGCGTCTATGACCAGGCGACATGGGAACAGGAGGACGCGCGCGATAATGAGTTCCT          414
```

```
V0-DWV-2                 nnnnnnnnnnnnnnnnnnnnnnnnnnnnnnnnnnnnnnnnnnnnnnnnnnnnnnnnnnnn        475
DWV-PA                   AACGGAACAATTAAACAATTTATATACTATTTATTCGATCGCTGAACGTTGTACGCGTCG        1414
DWV-304                  aacggaacaattaaacaatttatatactatttattcgatcgccgaacgttgcacgcgtcg        1440
Pupae-P0-DWV-2           AACGGAACAATTAAACAATTTATATACTATTTATTCGATCGCCGAACGTTGCACGCGTCG        467
Pupae-P1-DWV–Ext-2       AACGGAACAATTAAACAATTTATATACTATTTATTCGATCGCCGAACGTTGCACGCGTCG        474


V0-DWV-2                 nnnnnnnnnnnnnnnnnnnnnnnnnnnnnnnnnnnnnnnnnnnnnnnnnnnnnnnnnnnn        535
DWV-PA                   GCCTATCAAAGAGTACTCTCCTATATCAGTTTCGAATAGGTTTGCTCCACTGGAATCCCT        1474
DWV-304                  acctattaaagagcactctcctatatcagtttcgaataggtttgctccattggaatccct        1500
Pupae-P0-DWV-2           ACCTATTAAAGAGCACTCTCCTATATCAGTTTCGAATAGGTTTGCTCCATTGGAATCCCT        527
Pupae-P1-DWV–Ext-2       ACCTATTAAAGAGCACTCTCCTATATCAGTTTCGAATAGGTTTGCTCCATTGGAATCCCT        534


V0-DWV-2                 nnnnnnnnnnnnnnnnnnnnnnnnnnnnnnnnnnnnnnn-nnnnnnnnnnnnnnnnnnnn        594
DWV-PA                   CAAGGTCGAGGTCGGTCAAGAAGCAGGCGAATGTATATTTAAGAAACCTAAATATACGCG        1534
DWV-304                  caaagtcgaggtcggtcaagaagcaggcgaatgtatatttaagaaacctaaatacacgcg        1560
Pupae-P0-DWV-2           CAAAGTCGAGGTCGGTCAAGAAGCAGGCGAATGTATATTTAAGAAACCTAAATACACGCG        587
Pupae-P1-DWV–Ext-2       CAAAGTCGAGGTCGGTCAAGAAGCAGGCGAATGTATATTTAAGAAACCTAAATACACGCG        594


V0-DWV-2                 nnnnnnnnnnnnnnnnnnnnnnnnnnnnnnnnnnnnnnnnnnnnnnnnnnnnnnnnnnnn        654
DWV-PA                   CGTTTGCAAGAAAGTGAAGCGTGTTGCAACTCGCTTCGTTCGTGAAAAAGTTGTTCGTCC        1594
DWV-304                  cgtttgcaagaaagtgaagcgtgttgcaacccgcttcgttcgtgaaaaagttgttcgtcc        1620
Pupae-P0-DWV-2           CGTTTGCAAGAAAGTGAAGCGTGTTGCAACCCGCTTCGTTCGTGAAAAAGTTGTTCGTCC        647
Pupae-P1-DWV–Ext-2       CGTTTGCAAGAAAGTGAAGCGTGTTGCAACCCGCTTCGTTCGTGAAAAAGTTGTTCGTCC        654


V0-DWV-2                 nnnnnnnnnnnnnnnnnnnnnnnnnnnnnnnnnnnnnnnnnnnnnnnnnnnnnnnnnnnn        714
DWV-PA                   TATGTGTTCTAGATCCCCTATGCTATTATTTAAGCTTAAGAAAATTATTTATGATTTGCA        1654
DWV-304                  tatgtgttctagatctcctatgctattatttaagcttaagaaaattatttatgatttgca        1680
Pupae-P0-DWV-2           TATGTGTTCTAGATCTCCTATGCTATTATTTAAGCTTAAGAAAATTATTTATGATTTGCA        707
Pupae-P1-DWV–Ext-2       TATGTGTTCTAGATCTCCTATGCTATTATTTAAGCTTAAGAAAATTATTTATGATTTGCA        714


V0-DWV-2                 nnnnnnnnnnnnnnnnnnnnnnnnnnnnnnnnnnnnnnnnnnnnnnnnnnnnnnnnnnnn        774
DWV-PA                   CTTATATAGATTAAGAAAACAGATTAGGATGTTGAGACGTCAAAAACAGCGCGATTACGA        1714
DWV-304                  cttatatagattaagaaaacagattaggattttgagacgtcaaaaacagcgcgagtatga        1740
Pupae-P0-DWV-2           CTTATATAGATTAAGAAAACAGATTAGGATTTTGAGACGTCAAAAACAGCGCGAGTATGA        767
Pupae-P1-DWV–Ext-2       CTTATATAGATTAAGAAAACAGATTAGGATTTTGAGACGTCAAAAACAGCGCGAGTATGA        774


V0-DWV-2                 nnnnnnnnnnnnnnTAATCTGTTACAATTATCGAATCCGGTGCAGGCAAAACCAGAGAT        834
DWV-PA                   GTTAGAGTGTGTCACTAATCTGTTACAATTATCGAATCCGGTGCAGGCAAAACCAGAGAT        1774
DWV-304                  gttagagtgtgtcactaatctgttacaattatcgaatccggtgcaggcaaaaccagagat        1800
Pupae-P0-DWV-2           GTTAGAGTGTGTCACTAATCTGTTACAATTATCGAATCCGGTGCAGGCAAAACCAGAGAT        827
Pupae-P1-DWV–Ext-2       GTTAGAGTGTGTCACTAATCTGTTACAATTATCGAATCCGGTGCAGGCAAAACCAGAGAT        834
                                        **********************************************

V0-DWV-2                 GGATAACCCTAATCCAGGACCTGATGGCGAGGGTGAAGTTGAATTAGAAAAGGATAGTAA        894
DWV-PA                   GGATAACCCTAATCCAGGACCTGATGGCGAGGGTGAAGTTGAATTAGAAAAGGATAGTAA        1834
DWV-304                  ggataaccctaatccaggacctgatggcgagggtgaagttgaattagaaaaggatagtaa        1860
Pupae-P0-DWV-2           GGATAACCCTAATCCAGGACCTGATGGCGAGGGTGAAGTTGAATTAGAAAAGGATAGTAA        887
Pupae-P1-DWV–Ext-2       GGATAACCCTAATCCAGGACCTGATGGCGAGGGTGAAGTTGAATTAGAAAAGGATAGTAA        894
                         ************************************************************

V0-DWV-2                 TGTTGTTTTAACAACTCAGCGAGATCCTAGTACATCTATTCCAGCGCCGGTGAGCGTAAA        954
DWV-PA                   TGTTGTTTTAACAACTCAGCGAGATCCTAGTACATCTATTCCAGCGCCGGTGAGCGTAAA        1894
DWV-304                  tgttgttttaacaactcagcgagatcctagtacatctattccagcgccggtgagcgtaaa        1920
Pupae-P0-DWV-2           TGTTGTTTTAACAACTCAGCGAGATCCTAGTACATCTATTCCAGCGCCGGTGAGCGTAAA        947
Pupae-P1-DWV–Ext-2       TGTTGTTTTAACAACTCAGCGAGATCCTAGTACATCTATTCCAGCGCCGGTGAGCGTAAA        954
                         ************************************************************


V0-DWV-2                 ATGGAGTAGATGGACTAGTAATGATGTAGTAGATGATTACGCCACAATCACATCTCGATG        1014
DWV-PA                   ATGGAGTAGATGGACTAGTAATGATGTAGTAGATGATTACGCCACAATCACATCTCGATG        1954
DWV-304                  atggagtagatggactagtaatgatgtagtagatgattacgccacaatcacatctcgatg        1980
Pupae-P0-DWV-2           ATGGAGTAGATGGACTAGTAATGATGTAGTAGATGATTACGCCACAATCACATCTCGATG        1007
Pupae-P1-DWV–Ext-2       ATGGAGTAGATGGACTAGTAATGATGTAGTAGATGATTACGCCACAATCACATCTCGATG        1014
                         ************************************************************

V0-DWV-2                 GTATCAGATTGCTGAATTTGTTTGGTCGAAGGATGATCCATTTGATAAGGAGTTAGCACG        1074
DWV-PA                   GTATCAGATTGCTGAATTTGTTTGGTCGAAGGATGATCCATTTGATAAGGAGTTAGCACG        2014
DWV-304                  gtatcagattgctgaatttgtttggtcgaaggatgatccatttgataaggagttagcacg        2040
Pupae-P0-DWV-2           GTATCAGATTGCTGAATTTGTTTGGTCGAAGGATGATCCATTTGATAAGGAGTTAGCACG        1067
Pupae-P1-DWV–Ext-2       GTATCAGATTGCTGAATTTGTTTGGTCGAAGGATGATCCATTTGATAAGGAGTTAGCACG        1074
                         ************************************************************
```

Supplementary Figure S2. DWV polymorphisms in honey bees and mites in Experiment 2: "*Varroa*-mediated DWV transmission experiment". Electropherograms of direct Sanger sequencing of RT-PCR fragments corresponding to the DWV LP region were amplified using RNA extracted from bees and mites pooled according to their treatment (Experiment 2, Figs. 2 and 4). **(a)** Honey bee pupae P0-DWV$_2$, infected with a clone-derived DWV isolate served as the "source bees"; **(b)** *Varroa* mites V0-DWV$_2$, which acquired DWV from the "source bees"; **(c)** Honey bee pupae P1-DWV-Ext$_2$ infected with DWV strains transmitted by the *Varroa* mites fed first on the "source bees". Clonal DWV accumulated in the honey bee pupae and divergent DWV accumulated in the *Varroa* mites, as evidenced by the presence of double peaks indicated by arrows.

(a) Pupae P0-DWV$_2$ – clonal DWV

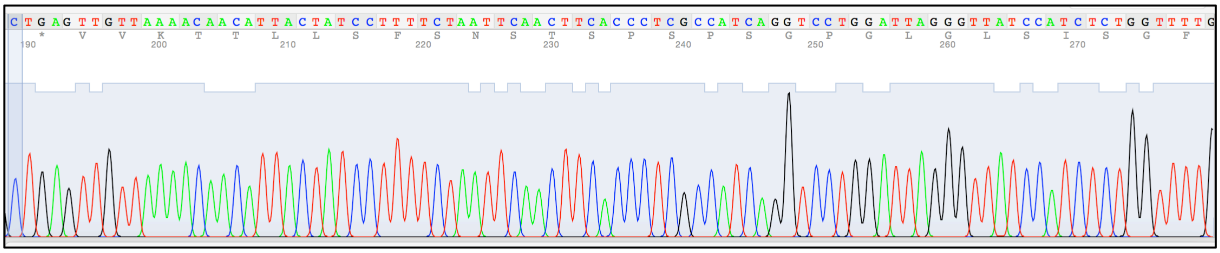

(b) *Varroa* mites V0-DWV$_2$ - polymorphic DWV

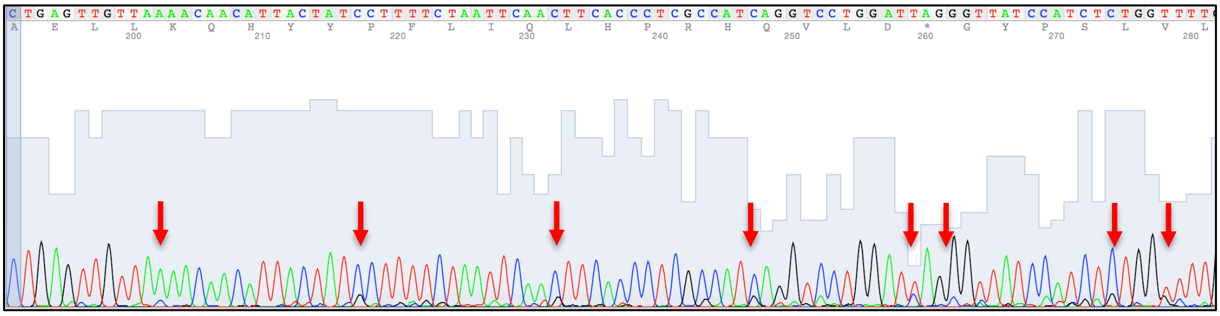

(c) Pupae P1-DWV-Ext$_2$ – clonal DWV

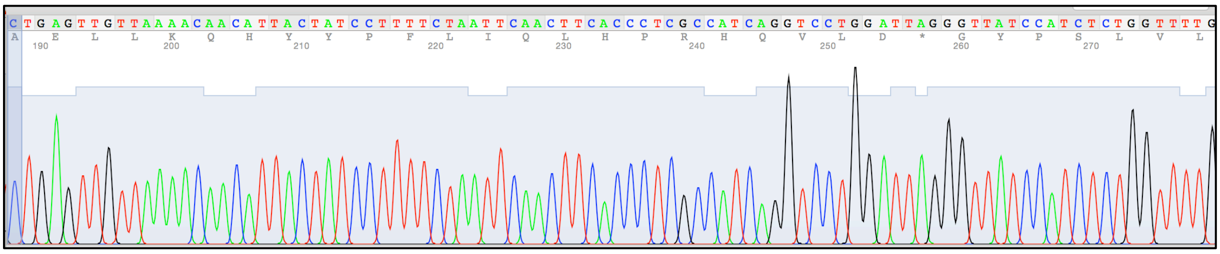

Supplementary Figure S3. Single stranded RNA degradation in disrupted honey bee hemolymph cells.

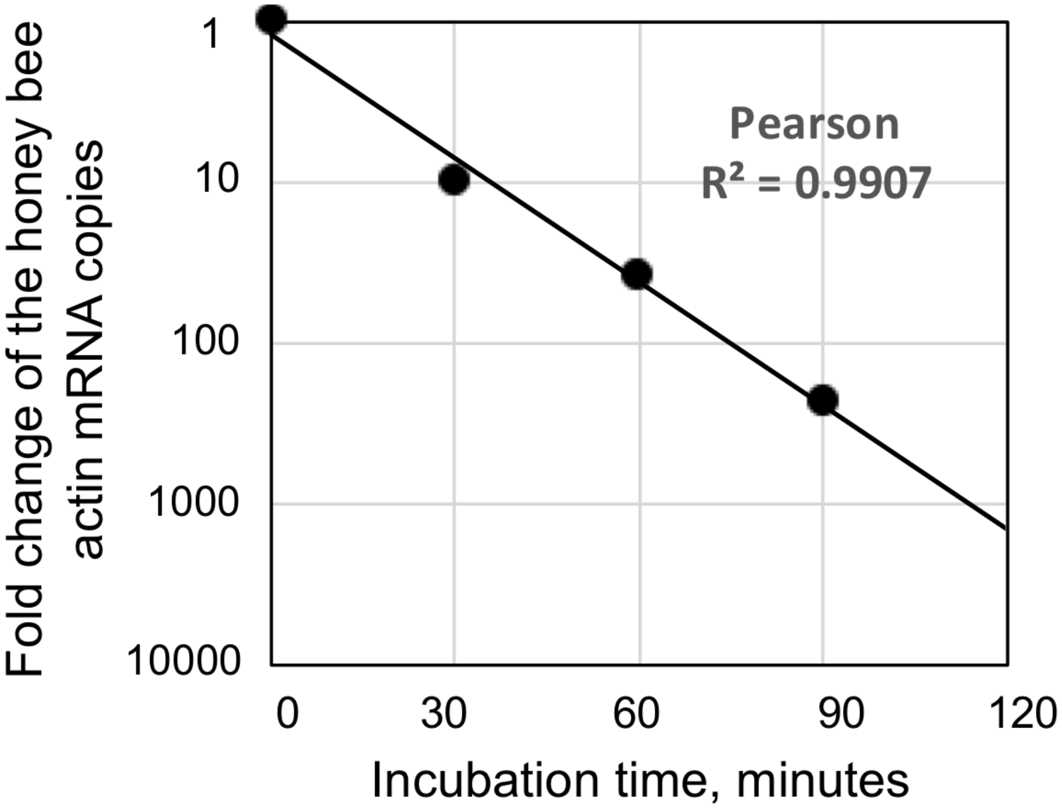

Supplement: Supplementary file 1 — Supplementary Information: Tables 1-4, Figures S1-S3. [file 41598_2019_47447_MOESM1_ESM.pdf]
